# Supplementary material for: Comparison of Genetic Susceptibility to Coronary Heart Disease in the Hungarian Populations: Risk Prediction Models for Coronary Heart Disease
Source: Genes (Basel). 2023 Apr 30;14(5):1033. doi: 10.3390/genes14051033 (PMC10218435; doi:10.3390/genes14051033)
Supplement: Supplementary file 1 [file genes-14-01033-s001.zip › genes-2315310-supplementary.pdf]

Supplementary File

Comparison of Genetic Susceptibility to Coronary Heart Disease in the Hungarian Populations: Risk Prediction Models for Coronary Heart Disease

Nayla Nasr [1,2], Beáta Soltész [3], János Sándor [1,4], Róza Ádány [1,4] and Szilvia Fiatal [1,*]

[1] Department of Public Health and Epidemiology, Faculty of Medicine, University of Debrecen, 4032 Debrecen, Hungary
[2] Doctoral School of Health Sciences, University of Debrecen, 4032 Debrecen, Hungary
[3] Faculty of Public Health, University of Debrecen, 4032 Debrecen, Hungary
[4] ELKH-DE Public Health Research Group, Department of Public Health and Epidemiology, Faculty of Medicine, University of Debrecen, 4032 Debrecen, Hungary
* Correspondence: fiatal.szilvia@med.unideb.hu

Supplemental Table S1: Single nucleotide polymorphism (SNPs) and relevant weights associated with CHD risk

No	SNPs	Nearest gene	Region/Band	RA	RAF			Weight	References
					1	2	3		
1	rs646776	SORT1	1p13.3	T	0.77	0.79	0.81	1.19	[1, 2, 3]
2	rs17114036	PPAP2B	1p32.2	A	0.92	0.89	0.91	1.17	[1, 2, 4]
3	rs11206510	PCSK9	1p32.3	T	0.82	0.81	0.81	1.15	[1, 4, 5]
4	rs17465637	MIA3	1q41	C	0.75	0.75	0.74	1.14	[1, 2, 4]
5	rs6725887	WDR12	2q33.1	C	0.13	0.14		1.17	[1, 3]
6	rs2306374	MRAS	3q22.3	C	0.10	0.18		1.12	[2, 4]
7	rs9818870	MRAS	3q22.3	T	0.15	0.17		1.15	[1, 6]
8	rs17609940	ANKS1A	6p21.31	G	0.79	0.81	0.75	1.07	[1, 2, 4]
9	rs9349379	PHACTR1	6p24.1	G	0.43			1.12	[1]
10	rs12526453	PHACTR1	6p24.1	C	0.67			1.10	[4]
11	rs12190287	TCF21	6q23.2	C	0.63	0.62		1.08	[1, 4]
12	rs3798220	LPA	6q25.3	C	0.01			1.47	[1]
13	rs10455872	LPA	6q25.3	G	0.07			1.70	[1]
14	rs11556924	ZC3HCL1	7q32.2	C	0.64	0.67	0.62	1.09	[1, 2, 4]
15	rs4977574	CDKN2B	9p21.3	G	0.55	0.43	0.46	1.29	[1,2, 4]
16	rs579459	ABO	9q34.2	C	0.22	0.21		1.10	[2, 4]
17	rs635634	ABO		T	0.20			2.05	[7]
18	rs1746048	CXCL12	10q11.21	C	0.86	0.84		1.17	[1, 2]
19	rs12413409	CYP17A1	10q24.32	G	0.90	0.92	0.89	1.12	[1, 2, 4]
20	rs964184	APOA5	11q23.3	G	0.13	0.14	0.13	1.13	[1, 2, 4]
21	rs2259816	HNF1A	12q24	T	0.35	0.37		1.08	[1, 6]
22	rs3184504	SH2B3	12q24.12	T	0.48	0.44		1.13	[1, 4]
23	rs4773144	COL4A1	13q34	G	0.41	0.40	0.44	1.07	[1, 2, 4]
24	rs2895811	HHIPL1	14q32.2	C	0.45	0.42	0.43	1.07	[1, 2, 4]
25	rs3825807	ADAMTS7	15q25.1	A	0.57	0.65		1.08	[1, 2]
26	rs12936587	RASD1	17p11.2	G	0.53	0.65	0.56	1.07	[1, 2, 4]
27	rs216172	SMG6	17p13.3	C	0.64	0.35	0.37	1.07	[1, 2, 4]
28	rs46522	UBE2Z	17q21.32	T	0.48	0.55	0.53	1.06	[1,2, 4]
29	rs1122608	LDLR	19p13.2	G	0.77	0.79		1.15	[1, 2]
30	rs9982601	KCNE2	21q22.11	T	0.13			1.20	[1]

SNPs: Single nucleotide polymorphisms, RA: Risk allele, RAF: Risk allele frequency The value of the risk allele frequency based on references.

Supplemental Figure S1. Haploview LD results in study populations:

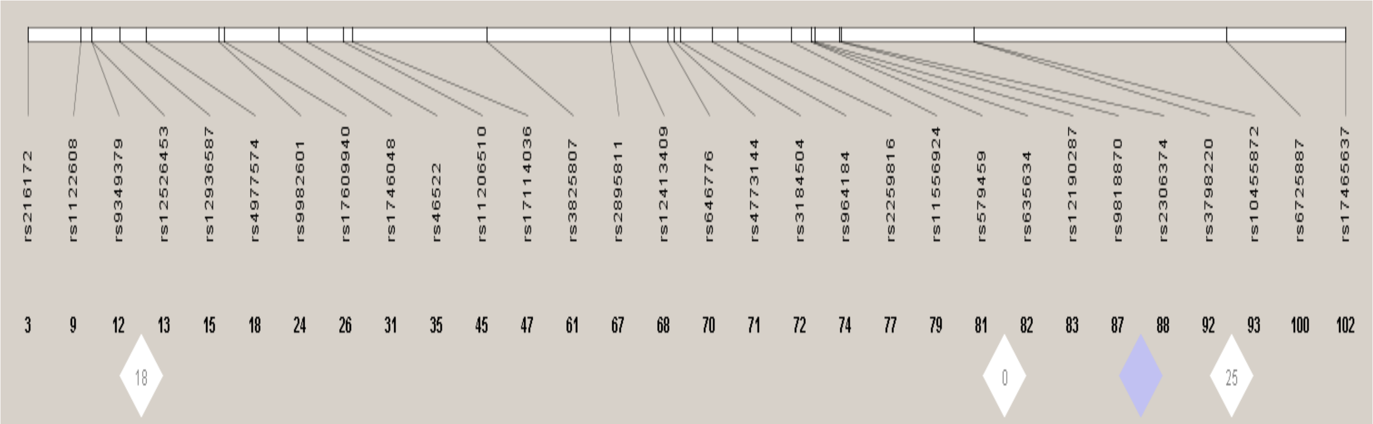

A. Haploview LD displayed the 30 SNPs for CHD risk among the Hungarian general populations.

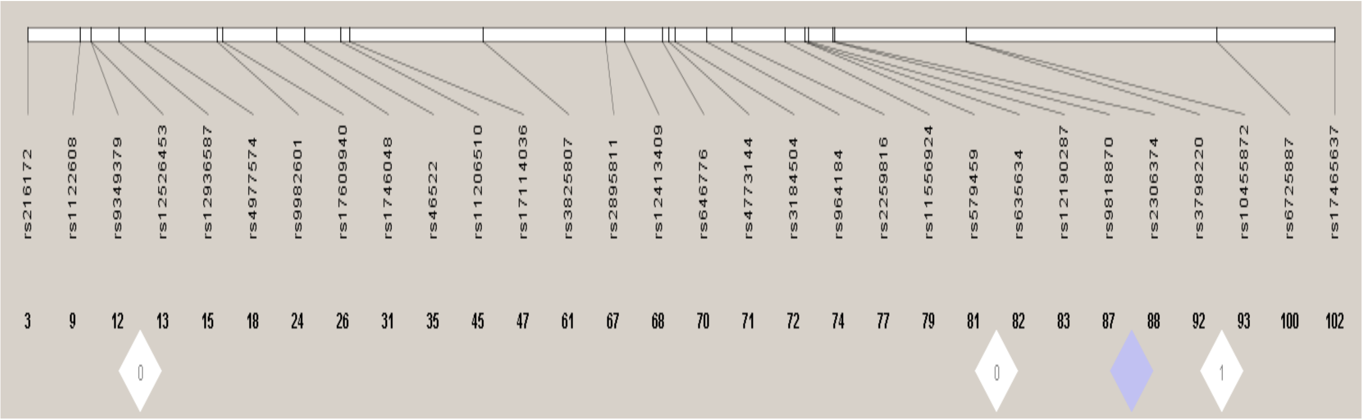

B. Haploview LD displayed the 30 SNPs for CHD risk among the Roma Hungarian Populations.

Supplemental Figure S1 A and B: Haploview LD display for the 30 SNPs predisposed to CHD in Hungarian general (n=279), and Roma Hungarian populations (n=279). The correlation based on genotyped alleles counts for expected linkage disequilibrium in Hungarian general (Fig A) and Roma Hungarian populations (Fig B) respectively. Alternative LD color scheme standard (D′)/LOD was used to measure the nonrandom association between the alleles in the 30 loci for CHD, there was no correlation identified among the SNPs.

Supplemental Table S2. The observed and expected frequencies of alleles according to Hardy Weinberg Equation for CHD variants in Hungarian general and Roma population.

No	SNPs ID	TEST	A1	A2	GENO	O(HET)	E(HET)	p-value
1	rs11206510	Total	T	C	405/132/21	0.237	0.263	0.023
		Roma	T	C	219/50/10	0.179	0.219	0.005
		Hungarian	T	C	186/82/11	0.294	0.303	0.558
2	rs17114036	Total	A	G	473/79/6	0.142	0.150	0.248
		Roma	A	G	236/41/2	0.147	0.148	0.694
		Hungarian	A	G	237/38/4	0.136	0.151	0.101
4	rs646776	Total	T	C	364/171/23	0.307	0.313	0.590
		Roma	T	C	191/77/11	0.276	0.292	0.410
		Hungarian	T	C	173/94/12	0.337	0.334	1
3	rs17465637	Total	C	A	255/247/56	0.443	0.436	0.772
		Roma	C	A	122/121/36	0.434	0.453	0.509
		Hungarian	C	A	133/126/20	0.452	0.418	0.200
5	rs6725887	Total	C	T	6/91/461	0.163	0.168	0.453
		Roma	C	T	2/43/234	0.154	0.154	1
		Hungarian	C	T	4/48/227	0.172	0.181	0.499
6	rs2306374	Total	C	T	12/94/452	0.169	0.189	0.022
		Roma	C	T	4/29/246	0.104	0.124	0.023
		Hungarian	C	T	8/65/206	0.233	0.248	0.331
7	rs9818870	Total	T	C	12/92/454	0.1649	0.186	0.011
		Roma	T	C	4/29/246	0.1039	0.124	0.023
		Hungarian	T	C	8/63/208	0.2258	0.243	0.221
8	rs9349379	Total	G	A	116/273/169	0.4892	0.496	0.798
		Roma	G	A	65/135/79	0.484	0.499	0.632
		Hungarian	G	A	51/138/90	0.495	0.490	1
9	rs12526453	Total	G	C	62/257/239	0.461	0.450	0.638
		Roma	G	C	31/127/121	0.455	0.448	0.894
		Hungarian	G	C	31/130/118	0.466	0.451	0.691
10	rs17609940	Total	G	C	470/84/4	0.151	0.151	0.782
		Roma	G	C	252/27/0	0.097	0.092	1
		Hungarian	G	C	218/57/4	0.204	0.206	0.776
11	rs12190287	Total	C	G	201/267/90	0.479	0.480	0.930
		Roma	C	G	83/142/54	0.509	0.495	0.717
		Hungarian	C	G	118/125/36	0.448	0.457	0.793
12	rs3798220	Total	C	T	0/8/550	0.014	0.014	1
		Roma	C	T	0/1/278	0.004	0.004	1
		Hungarian	C	T	0/7/272	0.025	0.025	1
13	rs10455872	Total	G	A	1/41/516	0.073	0.074	0.569
		Roma	G	A	0/11/268	0.039	0.039	1
		Hungarian	G	A	1/30/248	0.108	0.108	1
14	rs11556924	Total	C	T	261/241/52	0.439	0.430	0.694
		Roma	C	T	137/120/22	0.430	0.415	0.665
		Hungarian	C	T	124/125/30	0.448	0.443	1
15	rs4977574	Total	G	A	165/260/133	0.466	0.498	0.12
		Roma	G	A	92/134/53	0.480	0.480	0.716
		Hungarian	G	A	73/126/80	0.452	0.500	0.119
16	rs579459	Total	C	T	43/206/309	0.369	0.386	0.324
		Roma	C	T	19/97/164	0.348	0.363	0.509
		Hungarian	C	T	25/109/145	0.391	0.408	0.466
17	rs635634	Total	T	C	36/183/340	0.328	0.351	0.146
		Roma	T	C	10/87/182	0.312	0.31	1
		Hungarian	T	C	25/96/158	0.344	0.386	0.087
18	rs1746048	Total	C	T	362/174/22	0.3118	0.314	0.893

		Roma	C	T	177/88/14	0.3154	0.329	0.469
		Hungarian	C	T	185/86/8	0.3082	0.299	0.692
19	**rs12413409**	Total	G	A	399/130/29	0.233	0.280	**<0.001**
		Roma	G	A	189/69/21	0.2473	0.3187	**<0.001**
		Hungarian	G	A	210/61/8	0.219	0.2379	0.203
20	rs964184	Total	G	C	19/151/388	0.271	0.281	0.367
		Roma	G	C	12/84/183	0.301	0.312	0.565
		Hungarian	G	C	7/67/205	0.2401	0.248	0.627
21	rs3184504	Total	T	C	113/273/172	0.489	0.494	0.798
		Roma	T	C	33/138/108	0.495	0.464	0.303
		Hungarian	T	C	80/135/64	0.484	0.498	0.632
22	rs2259816	Total	T	G	94/272/192	0.488	0.485	0.931
		Roma	T	G	66/136/77	0.488	0.499	0.719
		Hungarian	T	G	28/136/115	0.488	0.451	0.232
23	rs4773144	Total	G	A	110/243/205	0.4355	0.486	0.015
		Roma	G	A	57/117/105	0.419	0.485	0.026
		Hungarian	G	A	53/126/100	0.452	0.486	0.267
24	rs2895811	Total	C	T	86/250/222	0.448	0.470	0.28
		Roma	C	T	37/118/124	0.423	0.451	0.291
		Hungarian	C	T	49/132/98	0.473	0.485	0.711
25	rs3825807	Total	A	G	167/276/115	0.495	0.496	1
		Roma	A	G	70/141/68	0.505	0.5	0.905
		Hungarian	A	G	97/135/47	0.484	0.484	1
26	rs216172	Total	C	G	49/242/267	0.434	0.424	0.618
		Roma	C	G	21/117/141	0.419	0.408	0.769
		Hungarian	C	G	28/125/126	0.448	0.438	0.785
26	rs12936587	Total	G	A	259/223/76	0.400	0.446	0.014
		Roma	G	A	150/108/21	0.387	0.393	0.763
		Hungarian	G	A	109/115/55	0.412	0.481	0.018
28	rs46522	Total	T	C	176/273/109	0.489	0.493	0.864
		Roma	T	C	90/139/50	0.498	0.490	0.808
		Hungarian	T	C	86/134/59	0.480	0.495	0.629
29	rs1122608	Total	G	T	315/201/42	0.360	0.380	0.221
		Roma	G	T	153/96/30	0.344	0.403	0.017
		Hungarian	G	T	162/105/12	0.376	0.356	0.401
30	rs9982601	Total	T	C	10/110/438	0.197	0.206	0.304
		Roma	T	C	3/42/234	0.151	0.157	0.439
		Hungarian	T	C	7/68/204	0.244	0.251	0.633

SNPs ID: SNPs identifier, TEST: Pearson's $\chi 2$ goodness-of-fit test, A1: minor allele code, A2: major allele code, GENO: genotype counts, O(HET): observed heterozygosity, E (HET: expected heterozygosity, RA(E/O): expected to observed ratios (corrected to uncorrected estimate ratios).

Supplemental Table S3. Odds ratio of CRFs and GRS for predicting CHD/AMI risk in the Hungarian populations.

CHDAMI	Hungarian general			Hungarian Roma		
	OR	p-value	95% CI	OR	p-value	95% CI
Age	1.082	**0.045**	1.002-1.170	1.018	0.529	0.963-1.077
Sex	1.501	0.560	0.383-5.878	2.753	0.103	0.815-9.297
HC-Med	5.793	**0.022**	1.289-26.039	3.446	0.055	0.974-12.189
HTN-Med	1.132	0.916	0.111-11.505	8.143	**0.001**	2.394-27.693
Smoking	0.934	0.927	0.217-4.018	1.230	0.740	0.362-4.178
GRS-T2	1.028	0.982	0.984-10.734	0.335	0.133	0.080-1.395
GRS -T3	2.185	0.505	0.219-21.759	0.755	0.695	0.185-3.085

Supplemental Table S4. Odds ratio of CRFs and wGRS for predicting CHD AMI risk in the Hungarian populations.

CHDAMI	Hungarian general			Hungarian Roma		
	OR	p-value	95% CI	OR	p-value	95% CI
Age	1.082	0.050	1.000-1.171	1.030	0.303	0.974-1.089
Sex	1.659	0.465	0.426-6.464	2.511	0.138	0.743-8.486
HC-Med	4.817	**0.036**	1.112-20.873	2.952	0.083	0.870-10.022
HTN-Med	1.271	0.837	0.129-12.484	**7.801**	**0.001**	2.394-25.405
Smoking	0.926	0.918	0.217-3.954	1.213	0.754	0.362-4.068
wGRS-T2	0.901	0.916	0.128-6.332	1.124	0.293	0.293-4306
wGRS -T3	1.291	0.775	0.224-7.430	0.731	0.165	0.165-3.246

Supplemental Table S5. Odds ratio of the CHD/AMI risk prediction model based on the CRFs plus DM in separate the study groups.

CHDAMI	Hungarian general			Hungarian Roma		
	OR	p-value	95% CI	OR	p-value	95% CI
Age	1.069	0.096	0.988_1.157	1.029	0.325	0.972_1.088
Sex	1.425	0.611	0.364_5.574	2.337	0.149	0.737_7.409
HTC	5.246	**0.027**	1.202_22.894	3.003	0.081	0.873_10.332
DM	4.337	0.081	0.833_22.576	0.990	0.988	0.260_3.761
HTN_Med	1.133	0.914	0.117_10.972	7.860	**0.001**	2.385_25.903
Smoking	1.146	0.859	0.257_5.114	1.263	0.705	0.377_4.232

Supplemental Table S6. Odds ratio of CRFs plus DM and GRS for CHDAMI risk prediction model among the study populations

CHDAMI	Hungarian general			Hungarian Roma		
	OR	p-value	95% CI	OR	p-value	95% CI
Age	1.071	0.086	0.990_1.157	1.019	0.512	0.963_1.079
Sex	1.387	0.642	0.349_5.516	2.744	0.104	0.814_9.246
HTC	5.856	**0.021**	1.306_26.269	3.515	0.054	0.976_12.653
DM	3.949	0.105	0.752_20.749	0.877	0.852	0.220_3.496
HTN_Med	1.033	0.978	0.105_10.135	8.258	**0.001**	2.413_28.260
Smoking	1.145	0.860	0.254_5.160	1.224	0.746	0.360_4.158
GRS -T2	1.005	0.997	0.095_10.586	0.330	0.129	0.079_1.383
GRS-T3	1.904	0.583	0.191_18.986	0.746	0.684	0.182_3.058

Supplemental Table S7. Odds ratio of CRFs plus DM and wGRS for CHDAMI risk prediction model among the study populations

CHDAMI	Hungarian general			Hungarian Roma		
	OR	p-value	95% CI	OR	p-value	95% CI
Age	1.068	0.104	0.987_1.156	1.030	0.315	0.973_1.090
Sex	1.457	0.592	0.368_5.776	2.514	0.138	0.743_8.510
HTC-Med	5.226	**0.031**	1.167_23.399	2.937	0.088	0.850_10.145
DM	4.327	0.088	0.804_23.295	1.034	0.962	0.266_4.020
HTN-Med	1.049	0.968	0.101_10.933	7.772	**0.001**	2.360_25.595
Smoking	1.146	0.859	0.257_5.110	1.214	0.754	0.362_4.070
wGRS-T2<br>wGRS-T3	0.822<br>1.102	0.846<br>0.916	0.114_5.912<br>0.182_6.694	1.131<br>0.731	0.860<br>0.681	0.289_4.425<br>0.164_3.253

Supplemental Table S8. Calculation of the AUC changes in the basic and updated models

**AUC changes for**	**Hungarian general**	**Roma**	**Combined population**
CRFs baseline vs. (CRFs+GRS) models:	ΔAUC=0.8346-0.8149=0.0197	ΔAUC=0.8549-0.8616= -0.0067	ΔAUC=0.8490-0.8479=0.0011
CRFs baseline vs. (CRFs+wGRS) models	ΔAUC=0.8160-0.814=0.0011	ΔAUC=0.8674-0.8616=0.0058	ΔAUC=0.8456-0.8479=-0.0023
CRFs +DM baseline vs. CRFs+DM+GRS models	AUC=0.8400-0.8299= 0.0101	ΔAUC=0.8534-0.8611= -0.0077	ΔAUC=0.8518-0.8525=0.0007
CRFs +DM baseline and CRFs+DM+wGRS models	ΔAUC=0.8400-0.8299= 0.0101	ΔAUC= 0.8670-0.8611=0.0059	ΔAUC=0.8479-0.8525=-0.0046

Supplemental Figure S2. Marginal relationship of CHD/AMI risk, age, and sex-adjusted for the Hungarian populations. The vertical axis shows the probability of CHD/AMI risk.

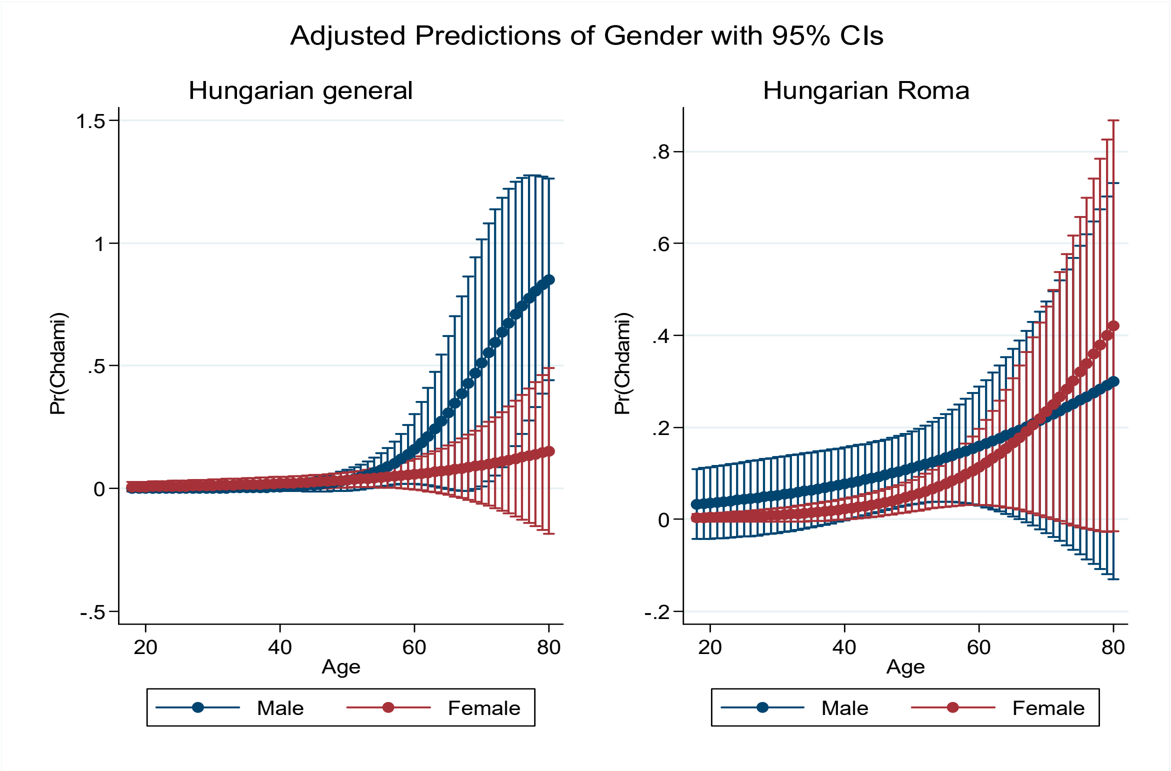

.

Supplemental Figure S3. Marginal plot analysis of the combined populations on CHD/AMI risk

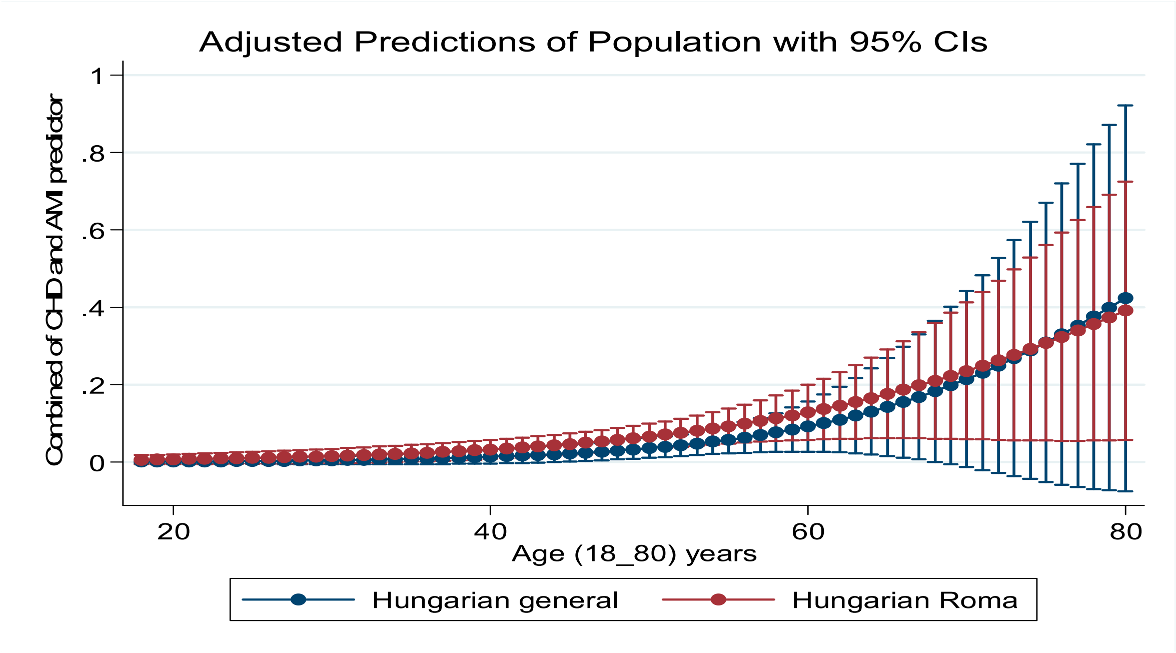

References

1. Mega, J.L.; Stitziel, N.O.; Smith, J.G.; Chasman, D.I.; Caulfield, M.J.; Devlin, J.J.; Nordio, F.; Hyde, C.L.; Cannon, C.P.; Sacks, F.M.; et al. Genetic risk, coronary heart disease events, and the clinical benefit of statin therapy: an analysis of primary and secondary prevention trials. *The Lancet* **2015**, *385*, 2264-2271, doi:10.1016/S0140-6736(14)61730-X.
2. Tikkanen, E.; Havulinna, A.S.; Palotie, A.; Salomaa, V.; Ripatti, S. Genetic risk prediction and a 2-stage risk screening strategy for coronary heart disease. *Arterioscler Thromb Vasc Biol* **2013**, *33*, 2261-2266, doi:10.1161/ATVBAHA.112.301120.
3. Kathiresan, S.; Voight, B.F.; Purcell, S.; Musunuru, K.; Ardissino, D.; Mannucci, P.M.; Anand, S.; Engert, J.C.; Samani, N.J.; Schunkert, H.; et al. Genome-wide association of early-onset myocardial infarction with single nucleotide polymorphisms and copy number variants. *Nat Genet* **2009**, *41*, 334-341, doi:10.1038/ng.327.
4. Schunkert, H.; König, I.R.; Kathiresan, S.; Reilly, M.P.; Assimes, T.L.; Holm, H.; Preuss, M.; Stewart, A.F.R.; Barbalic, M.; Gieger, C.; et al. Large-scale association analysis identifies 13 new susceptibility loci for coronary artery disease. *Nat Genet* **2011**, *43*, 333-338, doi:10.1038/ng.784.
5. McPherson, R.; Tybjaerg-Hansen, A. Genetics of Coronary Artery Disease. *Circulation Research* **2016**, *118*, 564-578, doi:doi:10.1161/CIRCRESAHA.115.306566.
6. Erdmann, J.; Großhennig, A.; Braund, P.S.; König, I.R.; Hengstenberg, C.; Hall, A.S.; Linsel-Nitschke, P.; Kathiresan, S.; Wright, B.; Trégouët, D.-A.; et al. New susceptibility locus for coronary artery disease on chromosome 3q22.3. *Nat Genet* **2009**, *41*, 280-282, doi:10.1038/ng.307.
7. Teslovich, T.M.; Musunuru, K.; Smith, A.V.; Edmondson, A.C.; Stylianou, I.M.; Koseki, M.; Pirruccello, J.P.; Ripatti, S.; Chasman, D.I.; Willer, C.J.; et al. Biological, clinical and population relevance of 95 loci for blood lipids. *Nature* **2010**, *466*, 707-713, doi:10.1038/nature09270.
